# Statistical Tools Applied in the Characterisation and Evaluation of a Thermo-Hygrometric Corrective Action Carried out at the Noheda Archaeological Site (Noheda, Spain)

**DOI:** 10.3390/s140101665

**Published:** 2014-01-17

**Authors:** Miguel Ángel Valero, Paloma Merello, Ángel Fernández Navajas, Fernando-Juan García-Diego

**Affiliations:** 1 Centro Asociado de la Universidad Nacional de Educación a Distancia en Cuenca, C/Colón 6, Cuenca 16002, Spain; E-Mail: mvalero@cuenca.uned.es; 2 CulturArts Generalitat (Instituto Valenciano de Conservación y Restauración de Bienes Culturales). Complejo Socio-Educativo de Penyeta Roja s/n., Castellón 12080, Spain; E-Mail: palomamerello@outlook.com; 3 Departamento de Física Aplicada, Universitat Politècnica de València. Av. de los Naranjos s/n., Valencia 46022, Spain; E-Mail: afnavajas@fis.upv.es; 4 Centro de Tecnologías Físicas. Universitat Politècnica de València, Av. de los Naranjos s/n., Valencia 46022, Spain

**Keywords:** microclimatic characterisation, temperature and relative humidity sensors, cultural heritage, preventive conservation

## Abstract

The Noheda archaeological site is unique and exceptional for its size, and the quality and conservation condition of the Roman mosaic pavement covering its urban *pars*. In 2008 a tent was installed as protection from rain and sun. Being of interest to characterise the microclimate of the remains, six probes with relative humidity and temperature sensors were installed in 2013 for this purpose. Microclimate monitoring allowed us to check relative humidity differences resulting from the groundwater level, as well as inner sensors reaching maximum temperatures higher than the outdoors ones as a consequence of the non-ventilated tent covering the archaeological site. Microclimatic conditions in the archaeological site were deemed detrimental for the conservation of the mosaics. Thus, in summer 2013, expanded clay and geotextile were installed over the mosaics as a corrective action. The outcomes of this study have proven the effectiveness of this solution to control temperature and relative humidity, helping to configure a more stable microclimate suitable for preservation of the mosaic.

## 1. Introduction

The Noheda archeological site can be found 0.5 km northwest of the village of Noheda (district of Villar de Domingo García), located 17 km from the city of Cuenca, in central Spain. It was declared “Heritage of Cultural Interest” by the Community Council of Castilla-La Mancha in 2012, mainly because of the mosaics discovered there. It is not only the late-antiquity era that contains evidence of human existence, although it is the most well documented period [1–4]. The last research carried out confirmed the existence of archeological structures dating to the 1st century A.D. under the late imperial Roman *villa*, but since the excavated surface is limited, its interpretation is currently impossible. Moreover, territorial analyses of the immediate surrounding area indicate intense human activities in these regions [5], showing evidence of the uninterrupted presence of a settlement from the prehistoric era until the Early Middle Ages.

Archeologists have known about these archaeological remains in the settlement for a long time [6–8], as the first evidence of the figurative mosaic was documented in the 1980s, thanks to the farming improvements that were taking place on the plot [9].

It was not until the end of 2005 when assessment of the archeological site began, quickly revealing its relevance. This realisation motivated the Community Council of Castilla-La Mancha to assume the responsibilities and financing of the site's excavation, allowing the research to proceed. For the past few years, the Provincial Deputation of Cuenca has been in charge of promoting the exploration work on the site.

The geographic location of the town in the peninsula, in the vicinity of cities from central Hispania, such as Ercávica, Valeria, Segóbriga and Complutum, demarcates a noticeable sort of crossroads, converting it into a natural path from south to north between mountain ranges, making it an area full of natural travel routes [10,11], which precisely follow some Roman roads.

To date, three areas of the villa have been exhumed: some structures belonging to the *pars rustica* (Sector III) of the rural complex, as well as a part of the *pars urbana*, consisting of a few rooms of the *balneum* (Sector II) and some of the rooms of the residential building (Sector I), although due to the insufficient amount of excavated surface we cannot determine which type of *villa* it belongs to [12,13].

This last sector is composed of several rooms of ample size, among them the so-called *Triconch* Room, called this way not only because of its impressive dimensions (290.64 m^2^) and its extraordinary pavements, but also for its complex architectural articulation and carefully decorated walls, comprising a skirting of marble tiles and a mural painting on the elevation. Its squared morphology, with exedras on three of the sides, allows us to ascribe it to the type of trifora rooms that were frequently built in the most luxurious residencial complexes dating from the end of the 3rd century A.D. [14]. These rooms have a *trichora* articulation, which are interpreted as *triclinia* [15–18], adapting the architectural form to the new tendencies of the spatial organisation for banquet guests, with beds available in a semicircle (the *stibadia*). In this room we find an exceptional mosaic with a conserved surface of 231.62 m^2^, mostly made of *opus vermiculatum* with a greatly varied range of colours, with coloured glass paste used for certain colours in a multitude of tones, including gold.

Noheda's remains are configured as a semi-confined archaeological site whose main remains of greatest interest are the Roman mosaics, which will be analysed in this work. As background, there are few studies of microclimatic conditions in semi-confined archaeological sites [19–21] and they are aimed at preventive conservation of wall paintings [19,20] and timber structures [21]. In [22] a list of 222 covered archaeological sites in Italy is available and an evaluation of the efficiency of the shelters is provided, but scores were calculated using qualitative criteria and not based on microclimate studies.

On the other hand, several works on preventive conservation of mosaics have been conducted in recent years. Calia *et al.* (2013) [23] made use of ground penetrating radar (GPR) prospection, infrared spectroscopy and thermo-gravimetric analyses, among others, to determine that the decay appeared to be due to the combined presence of water and soluble salts and had different effects depending on the characteristics of the materials in a mosaic in the crypt of St. Nicholas in Bari, (Southern Italy). Moropoulou *et al.* (2013) [24] used non-destructive techniques (GPR, infra-red thermography and fibre-optic microscopy) to evaluate the preservation state of the Hagia Sophia mosaics. In Alberghina *et al.* (2013) [25], spectrophotometric and colorimetric data were collated to study the possible variation of the chromatic coordinates, possibly due to the interventions for cleaning, consolidation and protection on the mosaic surface of the Roman “Villa del Casale” (Piazza Armerina, Sicily, Italy). Elsewhere, Faella *et al.* (2012) [26] studied the multidisciplinary activities performed in the Church of the Nativity in Bethlehem (Palestine) to achieve an accurate and reliable diagnosis of the construction and its mosaics. Temperature and moisture measures were performed as complementary methods.

However, nonspecific and exhaustive microclimatic studies of temperature and relative humidity (RH) were not performed in any of the aforementioned works. This paper concerns the installation of monitoring probes to provide useful data and the microclimatic characterisation of the preventive conservation actions undertaken to preserve the valuable and unique Roman mosaics of the archaeological site of Noheda, allowing us to take the results obtained as a starting point for future comparative and causal studies of the thermo-hygrometric conditions and conservation state and evaluate and quantify the effectiveness of the corrective action taken to control the temperature and relative humidity levels.

## 2. Materials and Methods

### 2.1. Monitored Room (Mosaics)

**Figure 1.**
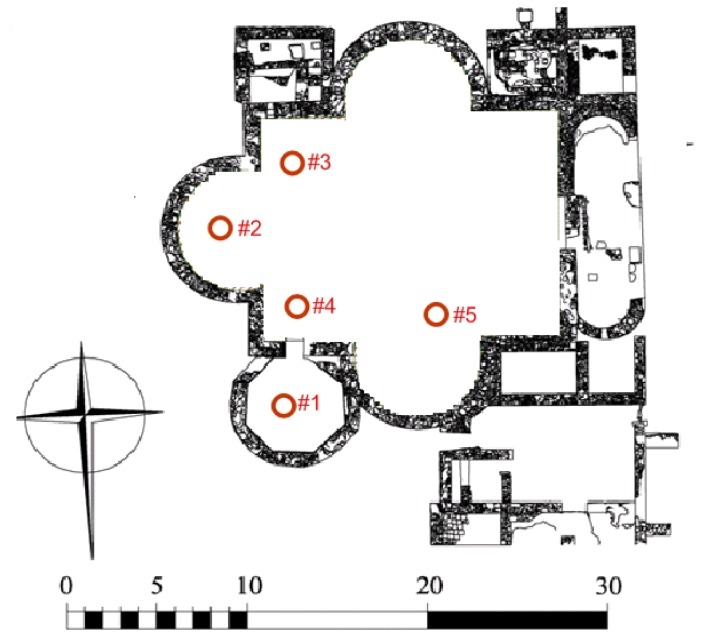
Plan of the archaeological site showing the location of sensors.

**Figure 2.**
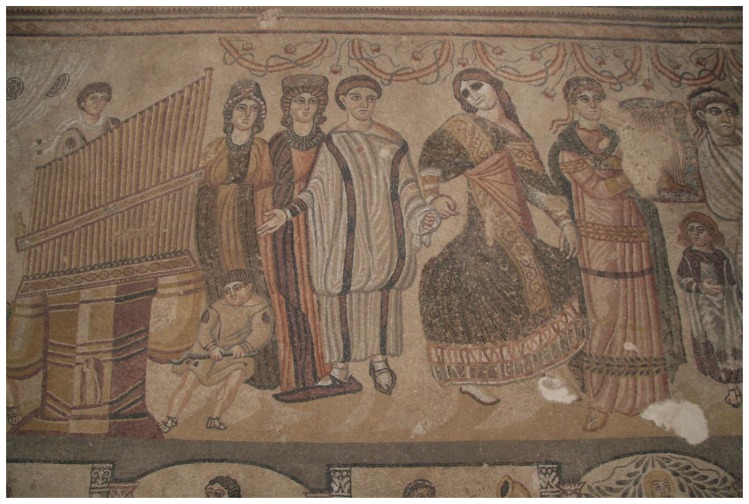
Mosaic detail.

The archaeological site of Noheda is comprised of two *pars* (the rustic and the urban). As mentioned above, it is in the urban *pars* (Figure 1) where residential building remains and mosaic pavement of exceptional conservation condition and measures were discovered (Figure 2), so this will be the object of study in this paper.

The mosaic consists of four technical layers: a levelling layer and a first level (statumen) consisting of river pebbles (14–18 cm in size); a second level, consisting of a white coating (8–10 cm thick) of lime mortar with stones, ceramics and high hardness fragmented bricks; a third level (5–6 cm thick) composed of lime, small stones and crushed ceramic which gives a reddish hue to the stratum. At the last layer we find the tesserae. The entire substrate of mortars exposed to the outdoors after excavation could be dispersed, so they were consolidated by successive impregnations with ethyl silicate (Estel 1000) dissolved in White Spirit D40.

In 2008–2009 a restoration campaign on the mosaic was conducted performing works of cleaning, consolidation (by injecting Primal SF 016 ER and using a solution of Paraloid B-72 to 3% in toluene for vitreous tiles) and stabilisation of the tapestry (adhesion of spare parts with Peoval 33).

Control of temperature and relative humidity is of great interest in preventive conservation of culture heritage [27]. There is a lack of consensus about the ideal or limit values of thermo-hygrometric parameters for optimum maintenance of mosaics. The UNI 10829 [28] and DM 10/2001 [29] international standards indicate reference values for the conservation of cultural heritage.

It is well known that buried cultural heritage can be found in prospections from 100% RH and at low temperatures (for example wrecks) as well as in very low humidity environments characterised by high temperatures (such as buried ruins in Egypt) and, in both cases, the good state of conservation is due to both parameters remaining constant. Thus, the most detrimental factor when studying RH and temperature is their variation. Extending this logic further, standard UNI 10829 [28] and DM 10/2001 [29] recommended maximum, minimum and maximum daily variations of temperature and RH. The admissible values suggested by this standard for stone mosaics (RH: 45%–60% and temperature: 15–25 °C) will be taken as a reference for the present work.

**Figure 3.**
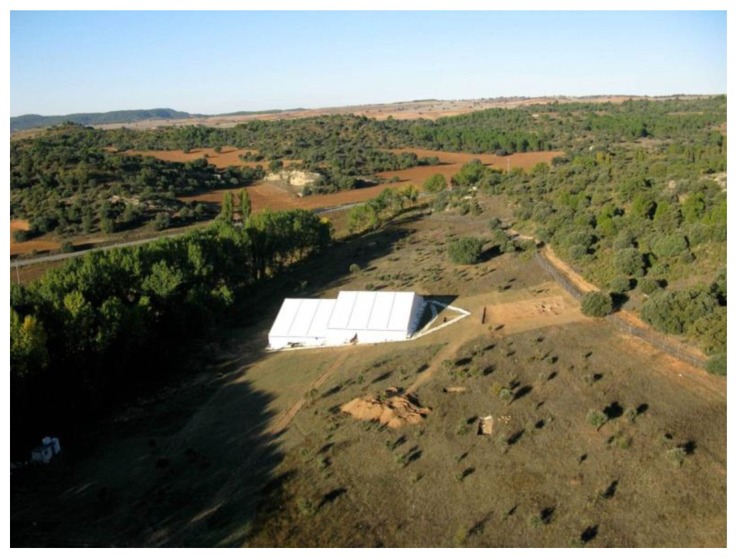
Aerial view of the site of the tent at Noheda showing the coverture that protects the mosaics.

With the aim of controlling the direct impact of sunlight, rain, wind and other erosion agents, the room containing the mosaic remains was covered with a non-ventilated waterproof canvas tent fixed to the ground with a concrete foundation supported over the plough line, so that it was not in direct contact with the buried stratigraphic layer. This covering (Figure 3) was installed on 15th April 2008 and is held in place every month of the year.

As a preventive conservation action, in the summer of 2013 (between 7 August 2013 and 12 August 2013) a solution for controlling temperature and relative humidity was implemented, consisting of a layer of expanded clay covering the mosaics and separated from them by a 0.4 cm thick geotextile. Expanded clay is a lightweight ceramic shell with a honeycombed core. The pellets are rounded in shape with a diameter of approximately 8–16 mm and an average dry bulk density of approximately 340 kg/m^3^. This is a common solution implemented in archaeology, designed to avoid the sudden variations in relative humidity and temperature using a porous insulating coating. Furthermore, this coating is easily removable if necessary, without damaging the remains.

### 2.2. Monitoring System

To check whether the corrective action was appropriate, a total of six probes were installed (each containing a data logger for temperature and a data logger for RH). The data loggers were purchased directly from the manufacturer (Maxim Integrated Products, Inc., Sunnyvale, CA, USA). These devices resemble button-type batteries, 17.4 mm in diameter and 5.9 mm thick. Each pair of RH and temperature data loggers was placed close together.

Each RH data logger (Datalog Hygrochron DS1923) contains a humidity sensor with an accuracy of ±5% [30]. Although this model can also record temperatures, it was decided to use independent devices (Datalog Thermochron DS1922L) to monitor the temperature [31], as the DS1922L provides the same accuracy (±0.5 °C) as DS1923, in order to enlarge the storage capacity of the monitoring system.

Sensors were calibrated prior to their installation through two calibration experiments (separated in time) in order to determine whether measurements from one or more sensors were biased compared to the average recorded by all sensors and correct the average sensor bias for all data, as detailed in [32].

The monitoring study started on 25 July 2013, when the six probes were installed at the archaeological site of Noheda (locations shown in Figure 1). All probes were placed attached to the inner top of a plastic bell without contacting the ground. The bell was supported on the floor by its open side, creating a microclimate similar to the microclimate the mosaics are exposed to. This procedure was selected because if the sensor is placed in direct contact with the mosaic it could saturate and measure 100% RH at all times, as well as metallic materials that might well possibly cause a chemical reaction that would harm mosaic tiles.

Location of sensors #1, #2, #3 and #4 was selected according to an area with a higher groundwater level, which was found by visual inspection of the darker mortar colour between the tesserae, in order to characterise the water vapour gradient of the site.

Sensor #6 was located on the outside of the tent of the archaeological site and in the shade (protected from direct impact of sunlight) as control sensor. Hereafter, this sensor will be referred to as “*out*”.

Sensors #1, #3 and #5 were placed over the mosaic tiles before placing the geotextile layer. Sensors #2 and #4 were placed above geotextile layer, obtaining a representation of the conditions configured by the implemented solution with and without installing a geotextile layer.

### 2.3. Data Analyses

The monitoring study began on 25 July 2013 and ended on 8 October 2013, resulting in a total period of 75 days. All data loggers were programmed to register one measurement every 30 min, which involves a total of 3,600 recorded values (*i.e.*, 75 days × 24 h/day × 2 data/h). Data corresponding to the three days from 7 August 2013 to 9 August 2013 were eliminated for sensors #1–#4, because these sensors were removed from the archaeological site those days to proceed with installation of the expanded clay and geotextile and the recorded data are not representative of the indoor climate of the archaeological site. Data recorded by the temperature and relative humidity monitoring system were analysed in this work by multivariate statistical techniques whose details are described below.

#### 2.3.1. Bivariate Plots

In [32], bivariate plots proved to be a simple technique that can be interpreted by visual inspection and provides similar results to cluster analysis. However, for confirmatory purposes cluster analyses were also performed in a previous step, also serving to give detailed information on each conglomerate.

#### 2.3.2. Cluster Analysis

In this paper, the squared Euclidean distance is used as a measure of similarity between observations and Ward's hierarchical method was applied. Aiming to quantitatively characterise the archaeological site before and after installation of the expanded clay and geotextile, two cluster analyses were performed (one for the period 25 July 2013–12 August 2013 and another for the period 13 August 2013–8 October 2013).

To measure the similarity between probes, the following variables were considered: mean daily maximum temperature and relative humidity, as well as the mean daily range (max-min) of temperature and relative humidity for each probe and period (before and after installing the expanded clay and geotextile).

For the case where there is a strong presence of multicollinearity between certain groups of variables, as a result of which underlying dimensions that are comprised of different numbers of variables are defined, including all the variables in the cluster analysis would give greater weight to that underlying dimension composed by a larger number of variables.

To avoid this problem, a correlation analysis of the four variables (*Tmax*, *RHmax*, *Tmax-min and RHmax-min*) was performed per period.

To ensure that the variables had equal weight in the analyses, they were performed for the standardised data because the variables were in different units of measure; results are presented in the original units because of their physical interpretation. All cluster analyses were performed using the SPSS 16 software [33].

#### 2.3.3. Mean Daily Trajectories

Mean trajectories summarise the information of the selected time period, avoiding excessively large plots and stationary periods and allowing simple comparison of different sensors or clusters [30]. Mean daily trajectories are calculated as the average of the data from each sensor per fraction of time (in this case, every hour) for the entire date range of interest. Temperature and RH mean daily trajectories were plotted in order to discuss the dissimilarities among probes and identify abnormal patterns.

#### 2.3.4. Contour Plots

Contour plots of water vapour pressure (mbar) were drawn up following the procedure described in [34].

## 3. Results and Discussion

### 3.1. Cluster Analyses

For data recorded prior to the installation of expanded clay, correlations between variables *Tmax*, *RHmax*, *Tmax-min* are all significant (*p*-value < 0.01) and with a correlation coefficient greater than 0.94. Thus, there is an underlying dimension that embraces a large percentage of variables (3/4). The cluster analysis is therefore performed with only the two RH variables, as they are enough to summarise the main similarities between probes.

For the data recorded after the installation of expanded clay, correlations between all variables are significant (*p*-value < 0.05) and high (correlation coefficient greater than 0.87). Thus, the four variables are considered in the cluster analysis in order to include the percentage of variability of each variable that is not explained by the others in the calculation of the clusters.

According to the study aim of identifying areas with singular behaviour inside the archaeological site, for data recorded before installing the expanded clay and geotextile a solution with 3 clusters was selected. The solution was chosen after analysing the results of solutions with higher and lower numbers of clusters (Table 1) and their distance matrix (Table 2).

As seen in Table 1, there are three clusters, whose Euclidian distance from the centres of all clusters is always greater than 21% of RH (Table 2). Cluster C3 is the cluster that contains the outdoor sensor and #5, and is characterised by the lowest maximum RH. Cluster C1 contains sensors #1 and #4, located at an area with a higher groundwater level and characterised by very high RH levels and the lowest daily variation of RH.

For data recorded after installing the expanded clay and geotextile, a solution with three clusters was selected (Table 3). The consistency of this solution can be determined by results shown in Table 4 (distance matrix). In Table 3 we can identify the probes comprised in each cluster.

Cluster C3, which contains the outdoor sensor, is especially different from cluster C1 (with a Euclidean distance of 39.99), as cluster C1 contains those probes placed in direct contact with the mosaic. Thus, once the expanded clay and geotextile is installed, the conditions present in the mosaic are distanced from those present outdoors, which are characterised by extreme thermo-hygrometric values and greater variability (Table 3), as indicated by the centre of C3.

### 3.2. Analyses of Relative Humidity Data

A detailed analysis of the relative humidity data was performed to determine the effects that the installation of expanded clay and geotextile has on this parameter and characterise in detail the aforementioned clusters in terms of RH, before and after the implemented measure.

**Figure 4.**
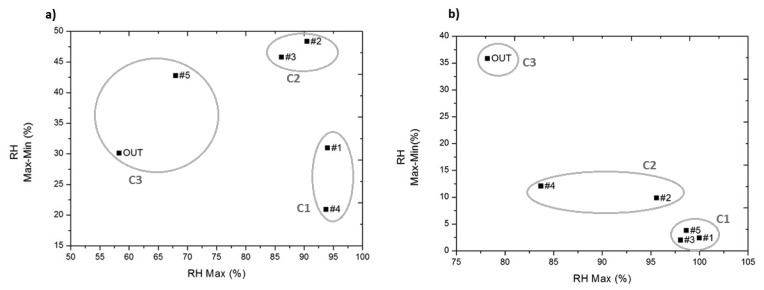
Bivariate plots of mean daily maximum of RH (RH Max) *versus* mean daily variation of RH (RH Max-Min). (**a**) From 25 July 2013 to 12 August 2013, from 00.00 to 23.59 h (864 data points/sensor); (**b**) from 13 August 2013 to 8 October 2013, from 00.00 to 23.59 h (2,688 data points/sensor).

**Figure 5.**
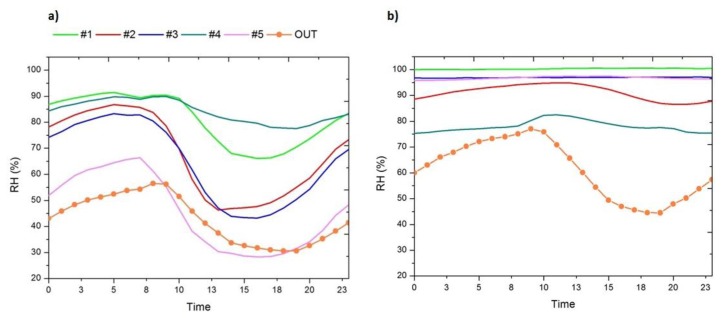
Mean daily trajectories of relative humidity, (**a**) for data recorded from 25 July 2013 to 12 August 2013; (**b**) for data recorded from 13 August 2013 to 8 October 2013.

As seen in Table 1, there are three clusters, each containing 1/3 of the sensors. Cluster C1 contains the sensors most similar to those of C2 (centre distance = 21.84% RH), but characterised by lower variability. Let us analyse the differences between these two clusters by comparing their bivariate plots (Figure 4) and mean daily trajectories (Figure 5).

**Figure 6.**
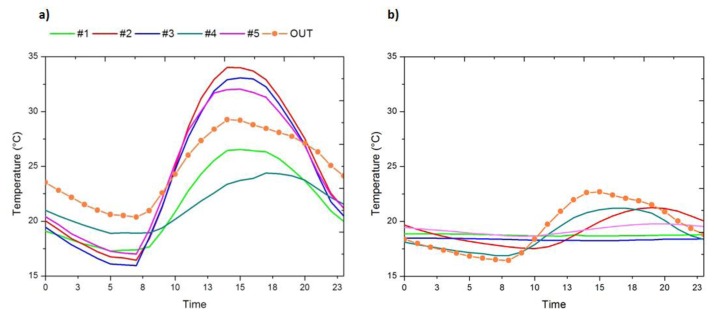
Mean daily trajectories of temperature, (**a**) for data recorded from 25 July 2013 to 12 August 2013; (**b**) for data recorded from 13 August 2013 to 8 October 2013.

As previously mentioned, prior to installation of the expanded clay and geotextile (Figure 4a), the similarity between sensor #5 and the *out* is given primarily in terms of the maximum RH, since sensor #5 is in an area with a lower groundwater level and reaches a mean daily maximum of 70% of RH (Figure 5a), and is characterised by higher daily variability of RH as a result of the extreme increase in temperature caused by the tent covering the archaeological site (Figure 6).

On the other hand, sensors contained in clusters C1 and C2 (#1, #2, #3 and #4) reach mean daily maximum values of RH higher than 85% as a consequence of their location in a wet terrain area of higher groundwater level; in particular, sensor #1 and #4 are characterised by reduced variability, reflecting RH increases by capillary action as a result of groundwater.

After installation of the expanded clay and geotextile, sensors inside the archaeological site reflect more constant mean daily trajectories (Figure 5b).

Among sensors installed inside the archaeological site, those sensors located under the geotextile (#1, #3 and #5), which represent the climatic conditions the mosaics are exposed to, reach values of RH higher than 95% and with a low daily variability (below the 10% of daily variability of RH recommended by the DM 10/2001 [29]). On the other hand, those sensors located above the geotextile (#2 and #4) have a higher variability and reach lower maximum values of RH, especially sensor #4.

**Figure 7.**
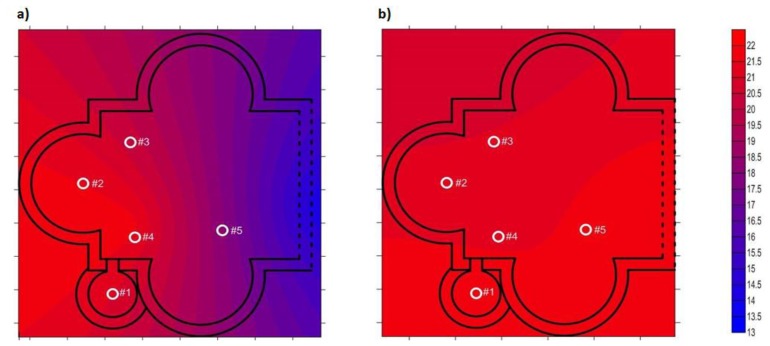
Contour plots of water vapour pressure (mbar), (**a**) for data recorded from 25 July 2013 to 12 August 2013 (864 data points/sensor); (**b**) for data recorded from 13 August 2013 to 8 October 2013 (2,688 data points/sensor).

Following, contour plots of water vapour pressure (Figure 7) are presented, allowing quick visual inspection of how this parameter has changed as a result of the installation of expanded clay and geotextile. Notice that for data recorded after the installation of expanded clay and geotextile, as we are interested in knowing the preservation conditions of the mosaic, only data recorded from sensors in direct contact with the mosaic tiles (#1, #3 and #5) were considered. From Figure 7, it follows that there is a horizontal gradient of water vapour pressure before the installation of expanded clay and geotextile (Figure 7a) which is directly related to an archaeological area having a greater contribution of groundwater. On the other hand, after the installation of expanded clay and geotextile (Figure b), water vapour pressure on the mosaic tiles was homogenised. This resulted in similar and quasi constant levels of RH in the sensors, matching the results obtained by cluster analysis and daily mean trajectories, and avoiding the formation of salt efflorescences because the cycles of relative humidity around the precipitation value were reduced.

### 3.3. Temperature Data Analyses

**Figure 8.**
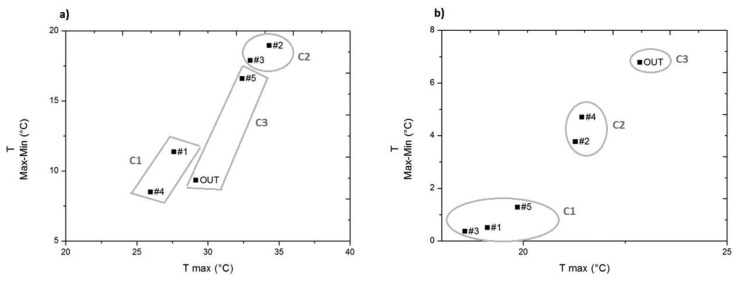
Bivariate plot of maximum temperature (T Max) *versus* mean daily variation of temperature (T Max-Min): (**a**) from 25 July 2013 to 12 August 2013, from 00.00 to 23.59 h (864 data points/sensor); (**b**) from 13 August 2013 to 8 October 2013, from 00.00 to 23.59 h (2,688 data points/sensor).

In the case of temperature, prior to the installation of expanded clay and geotextile, the two temperature variables (mean daily maximum and mean daily variation) help distinguish the three clusters obtained (Figure 8a). Thus, cluster C1 differs from C2 and C3, as it has lower daily maximum temperature values (10 °C).

On the other hand, C3 and C2, similar in their daily maximum temperature values, differ because C2 has a higher daily variability. Note that sensor #5, which had more similarities with the outdoors RH (Figure 4), in temperature seems to resemble more those sensors included in cluster C2 (Figure 8a), as can also be seen in the similarity of the mean daily trajectory of sensors #2, #3 and #5 in Figure 6a.

Note that the conglomerates of sensors that could be visually sensed in Figure 8a need not necessarily correspond to the cluster analysis results, as the variables included in the cluster analysis provide more information than the two temperature variables considered in the bivariate plot.

Prior to installation of the expanded clay and geotextile, sensors inside the archaeological site reflect the pattern of the outdoor sensor (Figure 6a) as a result of the heating effect of the cover. Furthermore, as the cover is non-ventilated, sensors #2, #3 and #5 reflect mean daily trajectories that reach maximum values higher (in 6 °C) than the maximum of the outdoor sensor.

After the installation of expanded clay and geotextile, all sensors located inside the archaeological site present maximum values and mean daily variability of temperature lower than the outdoor ones (Figure 8b). However, sensors in cluster C2 (#2, #4), placed above the geotextile, have a daily mean trajectory similar in shape to the outdoor one, but with lower maximum temperature values (Figure 6b).

Therefore, it can be said that the expanded clay and geotextile has control effects on temperature and these are particularly reflected by sensors installed directly on the mosaic (#1, #3 and #5) as a result of the insulating effect of the geotextile (Figure 6b).

## 4. Conclusions

The thermo-hygrometric monitoring system and data analysis methodology used in this work allowed the characterisation of the archaeological site of Noheda, of exceptional archaeological value, before and after a temperature and relative humidity control action performed on the mosaics.

Thus, statistical analyses have allowed us to identify, before installation of the expanded clay and geotextile, differences in RH between probes as a consequence of different groundwater levels (sensor #5 being similar to the outdoor one). On the other hand, for the same period of time, the inner sensors, which represent the conservation conditions of the mosaics, reach higher maximum temperatures (in 10 °C) than the outdoors as a consequence of the unventilated cover of the archaeological site.

As sensors installed above and below the geotextile have proven to give different results in terms of RH and T, then it can be concluded that the combination of both (geotextile and expanded clay) is the best solution, because over the geotextile the variations in RH and temperature were greater, while salt efflorescences are avoided because the cycles of increasing and decreasing RH necessary for their precipitation are broken.

After installing the expanded clay and geotextile, the results show that the solution implemented enables us to control temperature and RH, smoothing the mean daily variations of both parameters and creating a constant microclimate favourable for conservation of the mosaics.

The solution implemented for installation of the sensors, based on a waterproof and ventilated bell-shaped cover avoiding direct contact with the ground, is quite suitable for measuring the exchanges of the thermo-hygrometric parameters under study.

## Figures and Tables

**Table 1.** Results of cluster analysis for data recorded before installing the expanded clay and geotextile (from 25 July 2013 to 12 August 2013, from 00.00 to 23.59 h; 864 data points/sensor).

**Cluster**		**C1**	**C2**	**C3**
Sensor		1–4	2–3	5-OUT
Centres (%)	RH Max	93.91	88.33	63.15
	RH daily variation	25.94	47.05	36.43

**Table 2.** Matrix of Euclidian distances (in% of relative humidity (RH)) between the final cluster centres for cluster analysis of data recorded before installing the expanded clay and geotextile.

	**C2**	**C3**
C1	27.33	32.50
C2		21.84

**Table 3.** Results of cluster analysis of data recorded after installing the expanded clay and geotextile (from 13 August 2013 to 8 October 2013, from 00.00 to 23.59 h; 2,688 data points/sensor).

**Cluster**		**C1**	**C2**	**C3**
Sensors		1–3–5	2–4	OUT
Centres (%)	RH Max	99.45	89.65	78.21
	RH Daily variation	2.70	10.94	35.83
Centres (°C)	T Max	19.18	21.36	22.86
	T Daily variation	0.71	4.24	6.78

**Table 4.** Matrix of Euclidian distances between the final cluster centres for cluster analysis of temperature and RH data.

	**C2**	**C3**
C1	13.46	39.99
C2		27.55
